# The Effects of Combined Stress from pH and Microplastic-Derived Odours on the European Green Crab *Carcinus maenas*’s Olfactory Behaviour

**DOI:** 10.3390/ani15040464

**Published:** 2025-02-07

**Authors:** Hannah Ohnstad, Jonathan Burnett, Jörg D. Hardege

**Affiliations:** Biological Sciences, School of Natural Sciences, Hull University, Hull HU6 7RX, UK; h.ohnstad-2017@hull.ac.uk (H.O.); j.burnett-2015@hull.ac.uk (J.B.)

**Keywords:** *C. maenas*, behaviour, pheromone, polyethylene, attraction, pH, ocean acidification, feeding behaviour, stress

## Abstract

Ocean acidification (OA) associated with climate change is expected to lower the ocean’s pH by 0.5 units by 2100. Whilst associated effects such as coral bleaching and shell calcification are well documented, lesser-known impacts are the ‘invisible’ effects on animal sensory systems. Olfactory disruption impacts the behaviour towards chemical cues in many marine species, including crustaceans. We examine the effects of microplastic odour and additional stressors on the European green crab *C. maenas*. Using uridine diphosphate (UDP) and uridine triphosphate (UTP) as a sex pheromone bouquet, glutathione (GSH) as a food cue, and polyethylene (PE) as plastic odour, cues were mixed with carboxycellulose to create slow-release gels. Crabs were exposed to gels in seawater pH values of 8.2, 7.6, and 7.2. Crabs took longer to react to all odours in reduced pH conditions (pH 8.2 to pH 7.2, *p* = 0.0017). At a low pH, PE-exposed crabs exhibited attraction towards microplastic odour and changed behavioural responses by burying. The study confirms low pH as disruptive to olfaction and highlights that plastic derivatives can become more bioactive at reduced pH levels, potentially increasing the threat posed by microplastic pollution. Further research is required to determine the potential long-term impacts of the combined threat of microplastics and reduced pH in the environment.

## Simple Summary

The European shore crab, *Carcinus maenas*, also known as the European green crab, uses its sense of smell to detect prey, predators, and mating partners. Here, we examined how such crabs behave in environmental conditions that simulate the changes predicted by climate change. Crabs were exposed to female sexual odours, food smells, and various types of plastic leach outs. A decreased pH altered the crab’s behaviour towards food (Glutathione) and sex odour, reducing the animal’s response levels and increasing reaction times. Most interestingly, the crabs were more attracted to polyethylene (PE) odour in future ocean conditions, whilst males’ responses to female sex cues were especially reduced significantly. Response-level changes vary between the sexes, highlighting that understanding the effects of climate conditions on animal behaviour and choices is complicated and difficult to predict. That crabs become more attracted to plastic raises the question of what the bioactive chemicals are in PE that induce such a response, and this could demonstrate how climate change potentially increases the risks associated with plastic pollution in future oceans.

## 1. Introduction

### 1.1. Biodiversity in a Changing World

The marine environment plays a key role in global food security; marine species are valuable protein sources for human consumption; however, the development of technology with exponential human population growth has led to over-exploitation, with fishing pressures driving a serious depletion of ocean fishing stocks [1,2]. Biodiversity loss has increased over the last five centuries. Ecosystems are changing both locally and globally [3], with the IUCN red list reporting over 900 marine species lost during this period, including 229 molluscs, 80 fish, and 11 crustaceans [4]. Depleting resources lead to competition (both between species and interspecies)—a ‘survival of the fittest’ driving biodiversity extinction rates [3] and leaving over 1 million species facing extinction in the near future [5,6]. Much of this loss of biodiversity can be attributed to human-caused climate change.

Described as the change to global/regional climate patterns, climate change is largely driven by increased levels of atmospheric carbon dioxide (CO_2_) from increased burning of fossil fuels since the mid-20th century [7]. The demand for fossil fuels has continued to grow into the 21st century in order to meet the demands of the growing human population, with current atmospheric CO_2_ levels at 420 ppm (parts per million)—a rise of 145 ppm over the last 10 thousand years. Current predictions estimate that 1000 ppm could still be reached by the end of the century, even with intervention [8]. The impacts of climate change include sea level rise [9], ocean warming (OW) [10], unpredictable weather systems, an increase in extreme events, and other lesser-known problems such as ocean acidification (OA).

OA occurs when atmospheric CO_2_ dissolves into the water column at sea level and combines with H_2_O to form carbonic acid (H_2_CO_3_). Carbonic acid breaks down into bicarbonate (HCO_3_^−^) and a hydrogen ion (H^+^), causing seawater to acidify and a reduction in pH [11]. Whilst some areas of the marine environment already naturally have a lower pH, such as areas of natural upwellings, which may see pH falling as low as 7.2, and natural pH fluctuations within the seasons (lower pH in winter months due to CO_2_ dissolving more efficiently in lower temperatures), the process of OA has already seen global surface pH levels fall to 8.1 (current) from 8.2 (pre-industrial revolution), and it is predicted to cause acidification at 7.6 by 2100—an acidity increase of more than 100% [12].

### 1.2. Impacts on Olfaction

There have been recorded links between pH change and major threats to olfactory capacities and behavioural shifts in a range of marine species such as fish, crustaceans, polychaetes, molluscs, arthropods, chordates, and other macroinvertebrates [13,14,15]. *C. maenas*, the common shore crab also known as the European green crab, is a species which relies heavily on chemoreception as their predominant sense, which is used when exploring environments and in decision making, foraging [16,17], predator avoidance [16,18], and mating [16].

Olfactory receptors often detect single amino acids and peptides. The signal cues’ size and shape are a factor in signal binding and recognition, from which it is inferred that olfactory reception is vulnerable to structural changes, which can be affected by low pH and changes in salinity and temperature [19]. Roggatz et al. [20] examined the effects of OA and how it may alter chemical communication by changing the function and structure of peptide signalling molecules within *C. maenas*. The results showed that peptide signalling cues are susceptible to protonation in reduced pH conditions, altering the overall charge and charge distribution over the molecules and thus altering the signalling cues. Bioassays showed an impairment to the function of signalling peptides at a low pH. Modifications to the structure, charge, and function of signalling molecules can help explain altered behaviour in *C. maenas* and a range of other signalling systems when exposed to low pH [21]. This is one of the numerous studies that have highlighted that chemical cues play a huge role in the behavioural interactions of marine organisms [22,23,24,25], and in some cases, they are essential for species’ survival [26]. 

The greatest ocean pH changes can be observed at the surfaces, which is why most studies examine species that live within these habitats that are potentially better adapted to deal with fluctuations. However, Caldeira and Wickett [27] demonstrated that even minute changes to pH at the bottom of oceans could have major consequences for deep-dwelling species that are much more sensitive to environmental variation. There are very few studies that found any positive impacts of OA on the marine environment, except for marine algae, seaweeds (*Callophyllis lambertii*), and seagrass (*Posidonia oceanica*) benefitting from higher CO_2_ and allowing more photosynthesis to occur [28].

### 1.3. The Impacts of Microplastic

Plastic can now be found in nearly every environment from the poles to the equator [29]. Microplastics (MPs) are fragmented pieces of plastic measuring <5 mm in size [29], and they are usually sourced from car tyres, city dust, road markings, marine coatings, personal care products, and plastic pellets, with the most abundant source being synthetic textiles, accounting for over 35% of plastic in the world’s oceans. In 2018, 6.3 billion metric tonnes of plastic waste was produced globally, and it is believed that 79% is now in the natural environment or landfill sites [30]. MPs are now acknowledged as a persistent marine contaminant capable of absorbing hazardous chemicals and persistent organic pollutants (POPs) from the environment.

A key problem associated with MPs is that they become hosts for biofouling biota, which produce info-chemicals and biofilms, leading to the plastic’s projection of its own chemical signal. This is one reason some species are finding plastic an attractant [31,32,33]. In freshwater systems, a study on goldfish, *Carassius auratus,* was carried out by Shi et al. [34]; they investigated the impacts of MPs (polystyrene, PS) on olfactory-mediated behavioural responses, showing that behavioural responses were significantly hindered after 28-day exposure to PS. It is believed that exposure to MP could suppress the expression of genes encoding olfactory protein receptors, inhibiting ATPase transport [34].

Whilst a review of the literature reflects that a low pH is responsible for problems in a large range of marine species, very few studies have considered the combined effects of OA with an additional stressor (such as MPs, ocean warming, and toxicants). Manríquez et al. [35] investigated the combined effects of OA and OW on the mussel *Perumytilus purpuratus*. Mussels were exposed to different temperatures (15 and 20 °C) and different *p*CO_2_ (500 and 1400 μatm) for 10–14 weeks. The study showed that OA conditions led to increased oxygen consumption. However, the combination of OA and OW increased ATP demands and the use of carbohydrate reserves, demonstrating that more energy is used for other processes, which may negatively impact the species long term. MP pollution and OA are two consequences of anthropogenic activities. Within the natural marine ecosystem, simultaneous exposure to OA and MP pollution is becoming a much more likely scenario. Bertucci and Bellas [36] confirmed that MPs intensified the effects of OA on the larvae of sea urchins, *Paracentrotus lividus*; the combination of pH 7.6 and MPs significantly reduced larval growth in comparison to the controls. Solomon et al. [37] noted that even if current emissions targets are met a level of 1000 ppm, *p*CO_2_ may still appear. With pH levels continuing to fall and the quantity of plastic in marine environments on the rise, this combination could be more dangerous than just a single stressor.

### 1.4. The Studied Species and Aims of the Paper

*C. maenas* is attributed to high adaptability and tolerance to changing pH, temperatures, and salinities due to constant exposure to natural tide cycles. *C. maenas* are often used in behavioural studies, and they are highly studied in ecotoxicology experiments. They are commonly found and easy to measure and to determine their sex, and to mark individuals, making them a very suitable choice for bioassays [38]. Numerous studies have shown that the behavioural responses of an organism are heavily impacted by an individual’s characteristics [23]. Crab sex is known to alter the behavioural choices of certain chemical cues. For example, Richardson et al. [23] found sex-specific responses to a lowered pH when prey cues were present. Altered responses under future OA conditions have been observed, with delayed responses at pH 7.6. Males detected these cues much more rapidly than females, showing possible morphological or physiological differences between the sexes. Variables such as temperature, light intensity, water chemistry, noise, and background odours all potentially impact the behaviour of *C. maenas*, making it an interesting species for the purpose of this study [39,40]. Nevertheless, very few studies have considered the combined effects of ocean acidification upon olfactory cue driven behaviour with an additional stressor such as ocean warming or toxicants including microplastic [21,29,33,34].

The aims of this study are to establish how exposure to low pH affects the behaviour and responses of *C. maenas*, investigate whether exposure to MP odour impacts behaviour and reactions, and determine whether the combined stress of OA + MP will result in greater impacts to olfactory cue-controlled behaviours. The hypotheses are as follows: (1) the reduction in pH will significantly alter the olfactory responses of *C .maenas*, (2) microplastic odour will cause confusion within *C. maenas,* and (3) the combination of reduced pH and microplastic odour will have more severe impacts on the olfactory responses of *C. maenas.*

## 2. Materials and Methods

### 2.1. Experimental Animals

*C. maenas* (*n* = 170) were collected from Whitby, Northeast Yorkshire, at the end of May 2021 and stored in tanks of 6–8 crabs (males and females stored separately), with flow through natural seawater at a pH of 8.2 and at 15–20 °C. The crabs were fed twice weekly with frozen mussel (*Mytilus edulis*) meat during the testing period. *C. maenas* were acclimatised to aquarium conditions for one week before testing started. All individuals were marked for the duration of the study; carapaces were marked using non-toxic coloured nail varnish, each with a unique number and colour depending on the sex (previous studies have shown that nail varnish had no impact on the mortality rates of *C. maenas*) [41]. Markings lasted for the duration of the study and were reapplied if they began to wear off or if an individual moulted.

### 2.2. Chemicals and Bioassay Setup

Chemical cues were made by infusing natural odours of glutathione (food cue), female sex pheromones, and synthetic microplastic odours with carboxy–cellulose powder (medium-density sodium carboxymethyl–cellulose powder, Merck, Germany, produced by SIGMA ALDRICH, St. Louis, MO, USA, CAS number 9004-32-4) and mixed with artificial seawater at 34 ppm salinity to create a gel. In the case of control gels, purified water was added in place of a chemical odour. Pheromone gels contained uridine diphosphate (UDP) and uridine triphosphate (UTP) at a ratio of 4:1 [40] and at a concentration of 10^−3^ M. Glutathione (GSH) was the chosen food odour and was also made at a concentration of 10^−3^ M. Plastic odour was prepared by grinding 50 g of plastic pellets (*medium* density, SIGMA ALDRICH) per 230 mL of water in a blender for 5 min and then left for 48 h before being filtered. This was to remove the remaining plastic pellets from the conditioned water. The gels were then freeze-dried.

Preliminary testing was conducted to assess the cue dispersal rate and how long each odour lasted until it had entirely diffused and no longer elicited a behavioural response. These data can be found in Appendix A. To establish longevity, we added cues to the tanks and recorded the results until *C. maenas* stopped responding. This was repeated multiple times to establish average limits, and the results are shown in Appendix A. Red food colouring (TESCO own) was added to the flow and timed with respect to the duration it took to reach the opposite end of the tank to establish the flow rate. Odours took approximately 5 min to diffuse to the opposite side of the olfactometer and lasted approximately 2 h before requiring replacement.

Six experimental conditions were tested in Y-shaped olfactometers. These conditions were microplastic odour (PE) vs. GSH (food cue), microplastic odour vs. pheromone (uridine-di-phosphate, UDP + uridine-tri-phosphate, UTP), GSH cue vs. pheromone, microplastic odour vs. control, GSH vs. control, and pheromone vs. control. Each condition was tested at the current ocean pH (8.2), predicted ocean pH by the end of the century (7.6), and the pH found in upwelling areas (7.2). Within each condition, 40 repeats were carried out for all three pH values—making up a total of 720 bioassays.

### 2.3. Behavioural Assay Procedures

Two identical Y-shaped olfactometer tanks were used to carry out the bioassays. The tanks were as described by Ohnstad et al. [42] and made of grey plastic to limit reflections and light and shadows while testing. Artificial seawater was created at 35% salinity by mixing 35 g of aquarium salt per litre of deionized water and stirring well. Seawater pH was dropped to the required levels by bubbling CO_2_ through it until reaching the required levels. The water was stored in 114 L water butts under the Y-shaped olfactometers at a temperature of 15–20 °C. Water was pumped from the butts up into the olfactometers, entering each tank at the two tips of the Y and leaving the Y at the base [42]. The flow rate was set at 1 L per minute based on preliminary testing: 500 mL per minute entering through each tip of the tank, allowing chemical cues to diffuse at an equal rate and odours from previous tests to be removed from the tanks before each experiment. Olfactometers were filled to 12 cm depth, and the base of the tanks was covered in a layer of marine sediment (2.5 cm thick), which was washed thoroughly to remove any odours prior to the addition. Both olfactometers were set up identically, with the only difference being the seawater pH in each tank’s water butts. Once pH, temperature, light intensity, and salinity were measured, the freeze-dried gels were placed into silicone tea strainers, which were then placed at the tips of the Y-shaped olfactometers with flow running over them, allowing the cues to diffuse. The cues being used were placed blindly at either end of the Y and switched at random intervals to eliminate any chances of bias and anomalies that may have occurred.

The experiment was carried out in the summer reproductive season (July–September 2021) for summer-caught crabs that were sexually active. Responsiveness to sexual stimuli was tested as a positive control by exposing the males to female sex odours (UTP and UDP) and watching for mating behaviour. Once mating behaviours such as scraping and cradling were observed, the crabs were ready to be used. The behaviours that were looked are as described by Ohnstad et al. [42], who provide a detailed description of what those behaviours look like. In short, wafting is described as the rapid back-and-forth movement of the crab’s mouthpiece; grabbing is when the crab physically grabbed the cue; burying is when the crab buries itself in the sediment; flicking is recorded if the crabs’ antennules started rapidly flicking; non-visible is defined as when there were no visible behaviours seen; cradling was recorded if we saw crabs cradle the cue. Escape was recorded if the animals tried to climb the tank, etc. Defence is defined as if the claws were raised and touched when they reached out to the cue. For each test, an individual crab was placed in a holding basket at the base of the olfactometer and then left to acclimatise for 2 min. The basket was lifted after two minutes, and the timer started. The data recorded were the initial reaction time (seconds), which was marked by rapid antennular flicking; cue choice (cue/control/neither); and behavioural response (e.g., no visible reaction/cradled the cue/buried/wafted frequently, etc.). Each test was conducted for a duration of 5 min. This was repeated with 40 crabs at all three pH values for each experimental condition. On each experimental day, the individuals tested were taken from different holding tanks in the crab culture system on rotation from a total culture of 170 crabs, ensuring that individuals were allowed at least four days of rest before they were used again.

### 2.4. Statistical Analysis

All statistical analysis was carried out in R studio v1.3. Raw data were input to Excel spreadsheets with simple columns and then saved as a comma-separated value file (.csv), allowing it to be read in R studio prior to analysis. Some figures were created via Microsoft Excel. The full R script can be accessed in appendices. All data were tested for normality with the Shapiro–Wilk test before analysis. The data were not normal *p* ≤ 0.05 and were discontinuous, so non-parametric tests were chosen. Wilcoxon matched pair tests were performed to compare average initial reaction times at the three different pH levels to see if the pH change caused a significant difference in these results. Kruskal–Wallis tests were conducted to test differences in individuality data in order to establish if certain personality traits of individuals caused certain results to reoccur.

## 3. Results

### 3.1. Time to Respond to Cues

**Figure 1:**
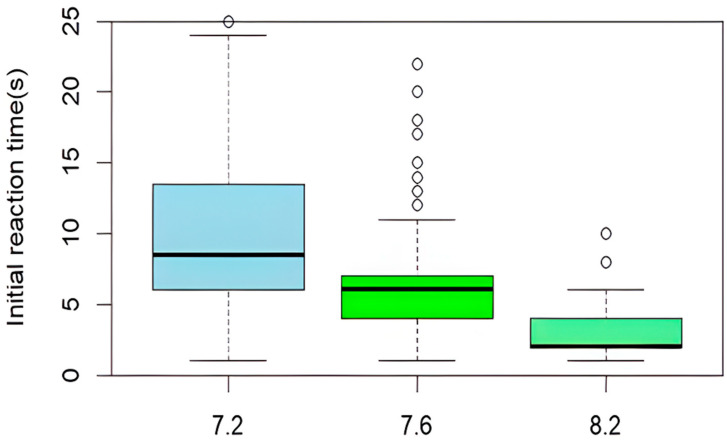
Combined results from all experiments showing average initial reaction time(s) at three pH levels: 7.2, 7.6, and 8.2. Initial reaction time was recorded as rapid antennular flicking. The lowest pH (7.2) saw a 9.9 s increase in reaction time over the highest pH (8.2); *n* = 240 in each pH. The whiskers on this plot show the majority of data, looking at the lowest and highest numbers; the dots are showing present outliers.

**Figure 2:**
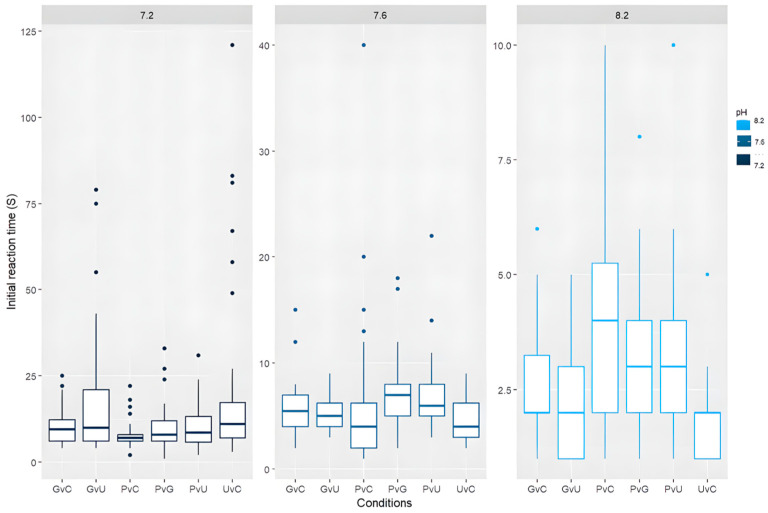
Initial reaction times (s) of *C. maenas* in each experimental condition under three different pH levels: 8.2, 7.6, and 7.2; *n* = 40 per condition. The pH is stated at the top of the figures. The reaction times are much larger in pH 7.2—most prominent in PE vs. glutathione and PE vs. control conditions. Conditions are GvC (glutathione vs. control), GvU (glutathione vs. UTP/UDP), PvC (PE vs. control), PvG (PE vs. glutathione), PvU (PE vs. UTP + UDP), and UvC (UTP + UDP vs. control). Notice the different scales in the y axis.

The average initial reaction time, as determined by antennular flicking, of *C. maenas* across each of the three pH levels was 2.79 ± 1.5 s (pH 8.2), 6.01 ± 4.2 s (pH 7.6), and 12.69 ± 10.98 s (pH 7.2)—a difference of 9.9 s between the highest and lowest pH. A paired *T*-test showed significance in all median initial reactions with a value of *p* < 0.05 [pH 8.2 and 7.6 t(11) = −9.045, *p* = 0.00001, between pH 8.2 and 7.2 t(11) = −6.33004, *p* = 0.0017, and between pH 7.6 and pH 7.2 t(11) = −3.60281, *p* = 0.001581], as seen in Figure 1. Whilst Figure 1 establishes that the crabs’ response time to odour cues is negatively correlated to pH, Figure 2 shows that the microplastic samples of PE vs. GSH and PE vs. control tests experienced significantly slower initial reaction times in pH 7.2 than other conditions. However, the PE vs. pheromone tests did not follow this trend.

Figure 2 shows the breakdown of initial reaction times in all six conditions tested, split into pH values of 8.2, 7.6, and 7.2. The initial reaction time in all paired conditions increases as the pH drops (Figure 2), following the trend shown in Figure 1. At a pH of 8.2, PE vs. control had the highest initial reaction time out of all conditions; this then drops to the lowest initial reaction time at pH 7.6 and 7.2, indicating quicker responses to this odour as pH falls in comparison to glutathione and UTP + UDP.

### 3.2. Cue Choice in Olfactometers

**Figure 3:**
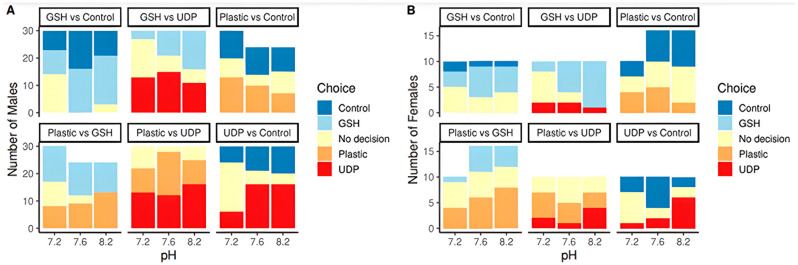
Number of male (**A**) and female (**B**) crabs selecting the cues (food, plastic, pheromone, and control) presented in each of the six paired conditions. Each column has three colours, two for each of the cues chosen and the yellow colour indicating that no cue was chosen (or that no decision was made).

Figure 3 shows a generalised plot of cues chosen in all six conditions, split into males and females for the choice made in the Y-shape olfactometer. The trends seen are a decrease in males choosing the GSH arm as the pH drops in the GSH vs. UTP/UDP trials examining food cues vs. sex pheromones.

There is an increase in no decision being made in males for most conditions as the pH falls. There is an increase in males choosing plastic as pH falls and a decrease in plastic being chosen when GSH was added, and pH fell. When looking at results in females, we can see plastic becoming more attractive as the pH falls, but more females chose plastic in pH 7.6 than pH 7.2. Again, as seen in males, the choice of plastic decreases with pH when GSH is present, and this could be due to added confusion from another odour. In females, there is a clear decrease in the choice of UTP + UDP as pH drops.

### 3.3. Behaviours Exhibited in Different pH Conditions

**Figure 4:**
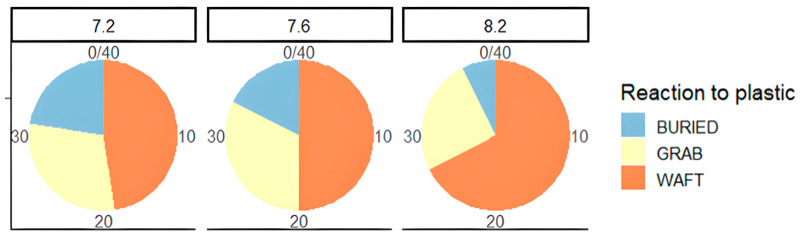
Number of crabs that were buried, grabbed the cue, or wafted in PE vs. control, *n* = 40.

The pie charts (Figure 4) look at the behaviours exhibited in PE vs. control at all three pH levels. Trends show the increase in burying as pH drops, wafting drops off and grabbing increases. Increased burying coinciding with decreased wafting shows that activity levels are reducing with falling pH, and this is potentially linked to increasing stress levels.

**Figure 5:**
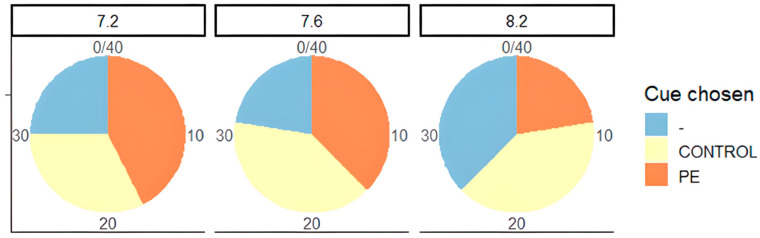
Percentage of crabs that chose the PE cue, control cue, or neither in each of the three pH conditions in PE vs. control, *n* = 40.

The second of these pie charts (Figure 5) looks at the cues chosen in PE vs. control, and it is split into all three pH levels. As pH falls, we can see more crabs choosing PE, showing that this odour is becoming more attractive. The number of crabs choosing the control and making no decision falls as the pH drops.

## 4. Discussion

The hypothesis that the combined effects of OA and microplastic odour as stressors result in greater impacts on olfactory detection in *C. maenas* was confirmed in several of the experimental conditions, particularly the PE vs. GSH (food) and PE vs. control tests, where initial reaction times were significantly slower at pH 7.2 compared to pH 7.6 and 8.2.

The crabs also displayed altered behavioural responses to cues (Figure 3), which could present consequences, such a greater risk of predation in the wild due to increased foraging time and less time sheltering; this type of negative behavioural impact has been observed in species such as the European sea bass *Dicentrarchus labrax*, which required 42% closer proximity to odours before detection in future OA conditions, lowering their chances of predator avoidance [43], and the shell-crushing crab *Acanthocyclus hassleri*, which was found to have decreased pinching strength as a result of OA, making them a less effective predator [44].

The results also demonstrate that OA alone alters olfactory senses, slowing the detection of cues without other stressors present. The literature shows that a lower pH affects the efficiency of stimulus receptor binding by altering the charge distribution on the stimulus molecule [20,43], leading to reduced responses and reduced prey detection in species such as *Pagurus tanneri*—a factor which could be considered a threat to the species long term [16,45]. In a study by Kim et al. [45], *P. tanneri* also displayed a similar delay in antennular flicking as was observed in this study; initial reaction times increased as pH fell (Figure 3), indicating that crabs take longer to recognise a cue in their environment under low pH/elevated CO_2_ conditions. A similar 2011 study looking at predatory reef fish found that elevated CO_2_ levels made larval fish more vulnerable to predation. However, Cripps et al. [46] also found predators spending 20% less time in elevated CO_2_ water containing prey odours, demonstrating that these cues are harder to detect—a positive factor for prey species. As prey detection will be lower, it is expected that predators such as *C. maenas* will spend less time within these areas [23].

Prey detection is only one factor affected by pH; however, *C. maenas* rely heavily on chemoreception for mating, especially in males that rely on detecting female pheromone odours during a very short opportunity period after the female moult [47]. Hayden et al. [48] demonstrated that males react to sex pheromones more frequently than food during the reproductive season (May–October). The research period of this study was during the *C. maenas* reproductive season, so males’ preferences for pheromones were expected. Males exhibited sexual behaviour during experiments, including searching activity, taking up cradling positions, and guarding behaviour [49] when UDP + UTP odours—nucleotides that are major components of female crab urine [50]—were present. Figure 3 demonstrates that the detection of pheromones was lowered when pH dropped, and this can be clearly seen in pheromone vs. control conditions. If males can no longer detect females accurately, *C. maenas* could struggle to reproduce, presenting potentially significant impacts on the reproduction of the species in affected ecosystems. This could also have serious implications for the growing field that uses pheromones in integrated pest management (IPM). Pheromones are a widely used method for IPM in terrestrial environments, particularly in the management of pests where the use of pheromone gels has effectively managed invasions [51,52]. Whilst terrestrial IPM is heavily studied and developed, Stebbing et al. [53] noted that there is currently a gap in the field of IPM research in the marine environment. The unique bouquets of pheromone gels used in this study (mixed into a gel using a carboxy–cellulose matrix as per Fletcher et al. [40]) are a technique that could potentially be used to help with the global invasion of *C. maenas* to manage populations and reduce their impact on ecosystems across its invasive range. However, as the pheromone and food cues used were not pH-stable, this study highlights that both environmental pH and microplastics in the ecosystem could negatively affect trapping efficiency and management efforts.

There were several limitations to the study; the first was that the crabs were stored in communal tanks, although males and females were stored separately. *C. maenas* are known to participate in dominance fights to establish hierarchy and social status within the tanks [54]. The winners of these fights are expected to react more often and more rapidly to chemical cues in comparison to the losers; in the case of UDP, 100% of the dominant males displayed full sexual behaviour after fights, whereas only 60% of the losers responded, showing increased latency in responses [55]. Dominant crayfish males keep their social status for a long period of time, and losers remember this [56]. As both winners and losers were present in this study, animal randomisation was used to ensure that the tests were unbiased. Nevertheless, this may help explain the large reaction time variability observed in the data. A further limitation was that the study was carried out in the summer during May–September, which are the reproductive months for *C. maenas* to carry out tests using sex pheromones. A study on Sea Bream, *Sparus auratus* [19], showed that amino acid responses fell only slightly in the presence of a feeding cue, and providing a male with a pheromone had deterring effects—if this study was to be repeated outside of reproductive season (i.e., winter), similar results in *C. maenas* may be observed [42].

Microplastic research often looks at ingestion, which can either be directly ingested as a food source or moves from the bottom of the food web upwards, starting with species such as zooplankton [57], with negative impacts for all species above via trophic transfer [58]. However, due to ethical concerns, this study used microplastic odour over raw pellets to avoid harm to *C. maenas*. Interestingly, only odour samples obtained from PE but not from PP and PVC induced behavioural changes in the crabs (Appendix A). To fully understand the impacts of microplastic odour, it will be key to examine which chemicals are represented in the PE samples. In hermit crabs, Greenshields et al. [33] described that Oleamide, a typical slipping agent additive in plastics is chemically related to oleic acid, a compound often described as the smell of death that is attractive to hermit crabs, *Pagurus bernhardus*. It remains to be examined if Oleamide is the bioactive compound leaching from PE that is responsible for attracting *C. maenas* and potentially also other marine scavengers.

In future studies, ocean warming (OW) with a predicted future temperature of +3 °C is an additional stressor occurring alongside OA and microplastic pollution that would be interesting to consider. The displacement of the Green Turtle *Chelonia mydas* has been observed in the ocean surface hotspots of the Southern Atlantic Ocean. *C. mydas* distributions are observed to change in relation to the sea surface temperature/warming of the water column [59]. *C. maenas* may see changes in seasonal residences as the temperature thresholds of shallower estuaries change, causing a shift to deeper ecosystems and a migration north, as seen on the west coast of the USA where the crab is a major invasive species. A study on the shrimp *Palaemon* spp. showed that exposure to OW leads to exhibiting riskier behaviour, such as foraging more actively for longer periods of time, even in the presence of a live predator [60]. Draper and Weissburg [61] noted that shifts in animal behaviour such as these will lead to long-term problems for prey species, benefitting predators. However, it is currently unknown how these factors will affect *C. maenas* in the long term. Testing to see if *C. maenas* eat less or grow slower in OA, MP, and OW conditions, or if mating becomes less successful over time, would be an interesting future study; however, as *C. maenas* have a relatively long reproductive season, conducting the study on another crustacean with a shorter reproductive cycle, such as *Gammarus pulex*, would allow the testing of multiple generations.

## 5. Conclusions

The conclusions of this study are that future OA/lower pH levels could cause significant alterations to the olfactory capacity of *C. maenas*, including lower response times, altered decision making, and difficulties in the detection of chemical cues. These data contribute to the growing field of climate change research observing the impacts of reducing pH on marine life, confirming that crustaceans such as *C. maenas* are negatively affected. Combined stressors, in this case, low pH and microplastic odour, had a greater impact on olfaction, suggesting that chemicals released from PE become more attractive at lower pH. The overall findings from this study demonstrate that OA will impact the key behaviours exhibited by *C. maenas* and create challenges for adaptation to future ocean conditions.

## Figures and Tables

**Figure A1:**
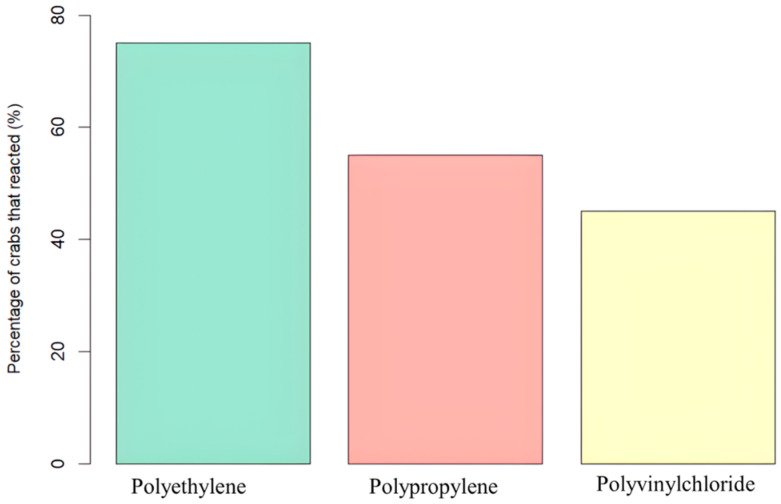
Percentage of *C. maenas* (*n* = 40 per assay set) that responded to plastic odour in a Y-shaped olfactometer (see Section 2); the expected null result is a 50:50 response.

## Data Availability

Data are available upon request.

## Appendix A

The appendix shows the responses of *C. maenas* upon exposure to odours of different types of plastic. Only polyethylene (PE) induced a behavioural response in the majority of crabs tested.

**Table A1:** This table shows preliminary results from flow rate testing. See Section 2.

Flow Rate	Time to Diffuse (minutes)
0.5 L/min	9.14
1 L/min	5.01
1.5 L/min	3.45
2 L/min	2.46
2.5 L/min	2.01
3 L/min	1.32
3.5 L/min	0.54
4 L/min	0.36
